# Impaired dynamic functional brain properties and their relationship to symptoms in never treated first-episode patients with schizophrenia

**DOI:** 10.1038/s41537-022-00299-9

**Published:** 2022-10-29

**Authors:** Wanfang You, Lekai Luo, Li Yao, Youjin Zhao, Qian Li, Yuxia Wang, Yaxuan Wang, Qian Zhang, Fenghua Long, John A. Sweeney, Qiyong Gong, Fei Li

**Affiliations:** 1 Huaxi MR Research Center (HMRRC), Department of Radiology, West China Hospital of Sichuan University, 610041 Chengdu, Sichuan P. R. China; 2 Research Unit of Psychoradiology, Chinese Academy of Medical Sciences, 610041 Chengdu, Sichuan P. R. China; 3 Department of Radiology, West China Second Hospital of Sichuan University, 610041 Chengdu, Sichuan P. R. China; 4 Department of Psychiatry and Behavioral Neuroscience, University of Cincinnati, Cincinnati, OH 45219 USA; 5 Department of Radiology, West China Xiamen Hospital of Sichuan University, 361021 Xiamen, Fujian China

**Keywords:** Schizophrenia, Biomarkers

## Abstract

Studies of dynamic functional connectivity (dFC) and topology can provide novel insights into the neurophysiology of brain dysfunction in schizophrenia and its relation to core symptoms of psychosis. Limited investigations of these disturbances have been conducted with never-treated first-episode patients to avoid the confounds of treatment or chronic illness. Therefore, we recruited 95 acutely ill, first-episode, never-treated patients with schizophrenia and examined brain dFC patterns relative to healthy controls using resting-state functional magnetic resonance imaging and a sliding-window approach. We compared the dynamic attributes at the group level and found patients spent more time in a hypoconnected state and correspondingly less time in a hyperconnected state. Patients demonstrated decreased dynamics of nodal efficiency and eigenvector centrality (EC) in the right medial prefrontal cortex, which was associated with psychosis severity reflected in Positive and Negative Syndrome Scale ratings. We also observed increased dynamics of EC in temporal and sensorimotor regions. These findings were supported by validation analysis. To supplement the group comparison analyses, a support vector classifier was used to identify the dynamic attributes that best distinguished patients from controls at the individual level. Selected features for case-control classification were highly coincident with the properties having significant between-group differences. Our findings provide novel neuroimaging evidence about dynamic characteristics of brain physiology in acute schizophrenia. The clinically relevant atypical pattern of dynamic shifting between brain states in schizophrenia may represent a critical aspect of illness pathophysiology underpinning its defining cognitive, behavioral, and affective features.

## Introduction

Schizophrenia is a severe and complex mental disorder marked by delusions, hallucinations, and cognitive impairments. Functional magnetic resonance imaging (fMRI) has helped identify the underlying neural mechanisms of these core illness features^1,2^. Viewed broadly, prior studies have indicated that schizophrenia is not caused by impairments of a small number of brain regions but by abnormal functional connections among widely distributed brain regions^3–5^. Much of our understanding of connectivity deficits in schizophrenia has been acquired from studies of static or average functional connectivity (FC) among brain regions. Because the functional organization of brain circuits is highly temporally dynamic, involving shifts among discrete brain states, investigating the dynamic changes may provide novel complementary information about abnormal brain neurophysiology in schizophrenia^6^.

Measures of dynamic functional connectivity (dFC) characterize the dynamic temporal patterns of brain connectivity over time. This is important, as it is the dynamic changes in activity states that are important for adaptive cognitive, affective, and behavioral processes. Dynamic connectivity studies have found that individuals with schizophrenia spend less time in a strongly and widely connected state (typified by strong, largescale, positive connectivity), correspondingly stay more in a weakly connected state (characterized by generally low strength of functional connectivity), and switched less frequently among discrete states in comparison with healthy controls (HCs)^7–10^. Patients have been reported to show increased dynamic variability within and between sensorimotor, visual, attention, and thalamic networks, and decreased intra-network and inter-network dynamic reconfigurations of default mode and fronto-parietal networks^11^. Another study reported increased dynamics of cross-network interactions among default mode network (DMN), salience network (SN), and central executive network (CEN) in schizophrenia^12^. These temporal and functional neuroimaging findings highlight the novel insights that can be obtained by investigating abnormal brain functional dynamics in schizophrenia.

Dynamic topology properties, which could describe quantitative topologies of brain regions in the context of complex whole brain systems based on graph theory^13^, have further provided useful information about the temporal dynamics of brain region interaction at a whole brain scale. One study using this type of network analysis reported an increment of dynamic nodal efficiency in the left frontal, right medial parietal, and bilateral subcortical areas in patients with schizophrenia^14^. In terms of whole-brain dynamic topological characteristics, reduced temporal fluctuation of clustering coefficient and global efficiency with impaired interaction among DMN subsystems have been reported in schizophrenia^15,16^, suggesting an important role of DMN in the pathophysiology of schizophrenia. Emerging research also indicates that dynamic connectivity alterations may be relevant for clinical symptoms in schizophrenia^8,14,17,18^.

Most studies investigating dFC have studied long-term ill and treated patients. This may be a limitation because of the potential confounding effects of antipsychotic medication and long-term illness on the brain^8–12,14,15,17,19^, which have been found to contribute to the dynamic changes observed over the progression of schizophrenia^20,21^. We are aware of only one study of acutely ill untreated patients, which recruited a relatively small group of first-episode, drug-naive patients to explore altered dFC strength in the mirror neuron system^18^. Larger studies of untreated first-episode patients are needed to evaluate dFC during acute psychotic illness without confounding the effects of drug treatment^22^.

We recruited acutely ill, never-treated, first-episode patients with schizophrenia and examined brain dynamic functional activity patterns relative to HCs. We hypothesized that: (1) patients would more often stay in a weakly connected state and (2) there would be an abnormal dynamic topology of regional brain properties in schizophrenia. Based on previous resting-state network meta-analysis^4,23^, we predicted alterations in DMN^11,12,16^, ventral attention network (VAN) engaged in processing of salience^24^, visual network (VN) associated with visual processing^25^, and sensorimotor network (SMN) involved in sensory and auditory perception^26^. We also predicted that altered temporal properties would correlate with patients’ clinical symptom severity. In addition, to evaluate dFC features for understanding illness biology at the level of individual patients, we conducted a supplementary machine learning study to identify features that most consistently were useful for identifying schizophrenia patients.

## Results

### dFC state characteristics

**Fig. 1:**
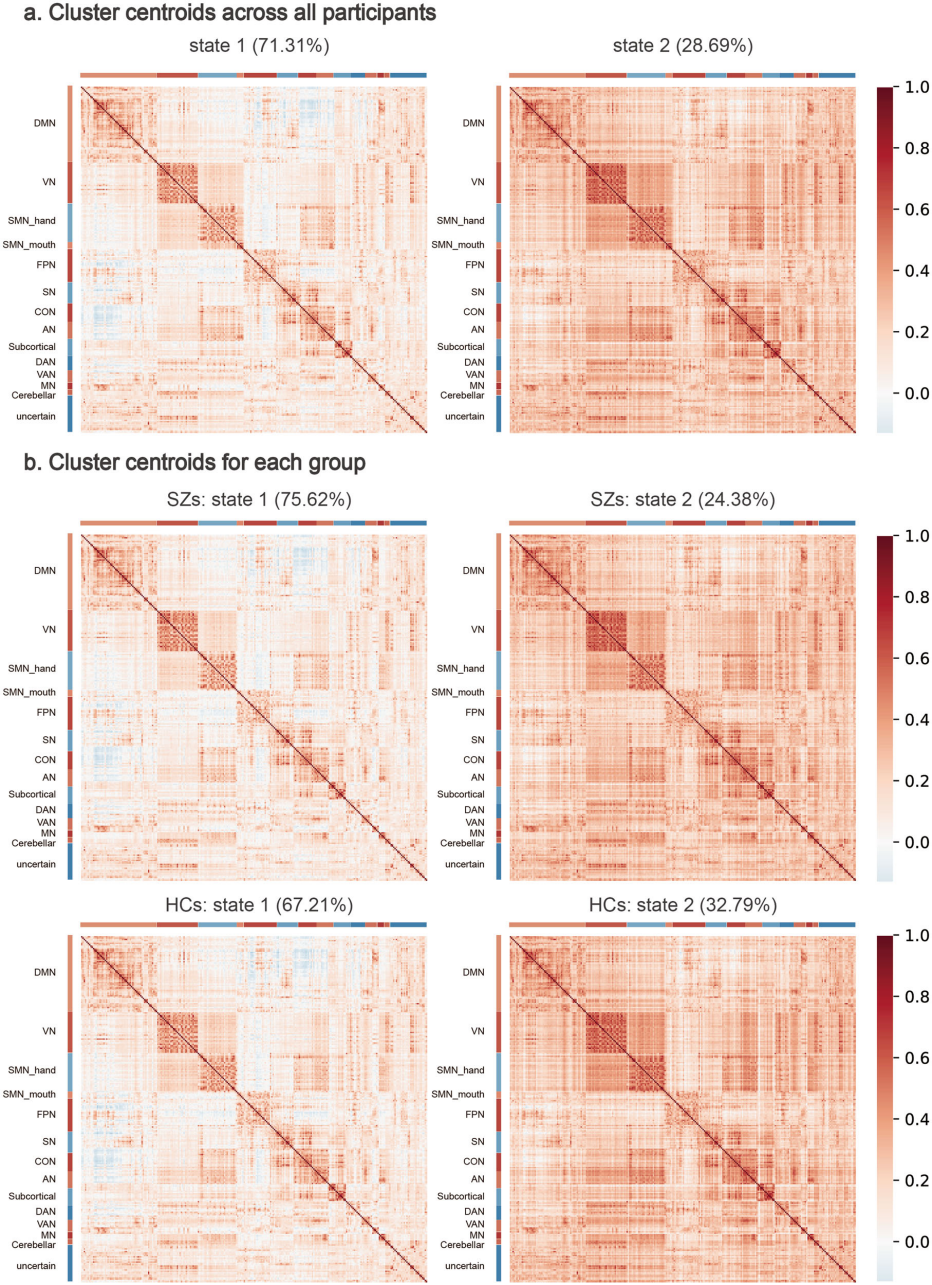
Cluster centroids of reoccurring dynamic functional connectivity (FC) patterns in the main analysis. **a** Cluster centroids for each state across all participants. **b** Cluster centroids for each state for each group. The value of each cell in the FC matrix is the Pearson correlation coefficient between two brain regions. The color bar shows the strength of FC between two nodes (warm color, positive FC; cool color, negative FC). DMN default mode network, VN visual network, SMN_hand/mouth sensorimotor hand and mouth networks, FPN fronto-parietal task control network, SN salience network, CON cingulo-opercular task control network, AN auditory network, DAN dorsal attention network, VAN ventral attention network, MN memory retrieval network, SZs schizophrenia patients, HCs healthy controls.

There were 95 never-treated first-episode schizophrenia and 100 matched HCs scanned and included in analyses (Table 1). Our first analysis was conducted to empirically define identifiable discrete brain states to analyze state change metrics. Two recurring brain functional states were defined by cluster analysis based on the similarity of the dFC correlation matrices. Their centroids are shown in Fig. 1 based on a window size of 22 times of repetition (TR). State 1 was characterized by weak connectivity both within and between networks, with negative couplings between DMN and other networks and between fronto-parietal task control network (FPN) and SMN, VN, cingulo-opercular task control network (CON), and auditory network (AN). State 2 had stronger and mostly positive connectivity in general, including strong and positive dFCs within and between SMN and VN.

**Table 1:** Demographic and clinical characteristics of patients with schizophrenia and healthy controls.

	Patients (*n* = 95)	Controls (*n* = 100)	*χ*^2^/*t* value	*P* value
Sex (number)	41M, 54F	47M, 53F	0.290^a^	0.590
Age (years)	24.87 (7.40)	25.74 (7.43)	0.815^b^	0.416
Education (years)	12.83 (2.77)	12.95 (3.15)	0.278^b^	0.781
Duration of illness (months)	7.18 (8.54)	–	–	–
*PANSS symptom ratings*				
Total ratings	89.11 (16.77)	–	–	–
Positive symptom ratings	25.55 (6.37)	–	–	–
Negative symptom ratings	17.62 (7.61)	–	–	–
General symptom ratings	45.94 (10.01)	–	–	–

The data are given by the mean (standard deviation).

*M* male, *F* female, *PANSS* positive and negative syndrome scale.

^a^Chi-square test.

^b^Two-sample *t*-test.

**Fig. 2:**
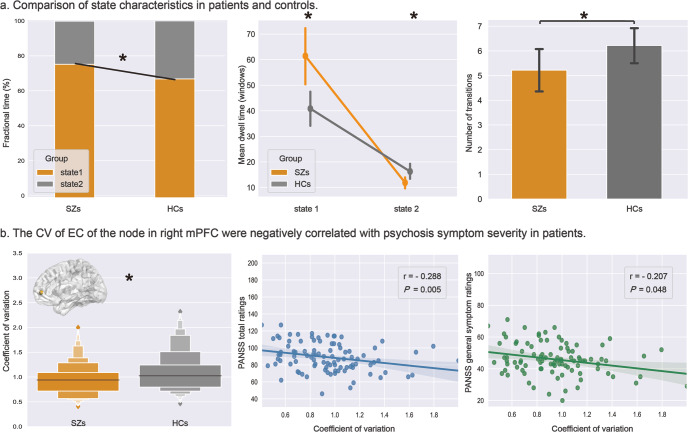
Altered dynamic properties between groups and correlations between dynamics and symptoms in patients. **a** Patients with schizophrenia (SZs) showed altered fractional time and mean dwell time in both states, and decreased transitions between states relative to healthy controls (HCs). **b** The coefficient of variation (CV) of eigenvector centrality (EC) of the right medial prefrontal cortex (mPFC) was decreased in SZs than HCs and was negatively correlated with psychosis symptom severity in patients. * Indicates false discovery rate corrected *P* value < 0.05.

The frequency of occurrence of the hypoconnected state 1 (71.3%) was higher than that of hyperconnected state 2 (28.7%) across all participants (Fig. 1a). The fractional time of brain function in state 1 was significantly higher in patients relative to controls after false discovery rate (FDR) correction (patients 75.6 ± 25.1%, HCs 67.2 ± 27.0%, FDR-*P* = 0.017). Accordingly, patients spent less time in state 2 of high connectivity compared with controls (patients 24.4 ± 25.1%, HC 32.8 ± 27.0%, FDR-*P* = 0.017). We also found that patients demonstrated longer mean dwell time in state 1 (patients 61.5 ± 52.7, HCs 41.0 ± 35.0, FDR-*P* = 0.002) and reduced mean dwell time in state 2 relative to HC (patients 12.2 ± 10.2, HCs 16.6 ± 15.6, FDR-*P* = 0.017). The number of transitions between states was lower in schizophrenia patients than HCs (patients 5.2 ± 4.3, HCs 6.2 ± 3.7, FDR-*P* = 0.044) (Fig. 2a). No significant correlations between these global state characteristics and clinical ratings were observed in patients. In addition to the primary analysis using 22TR window sizes, we did a secondary analysis with 30TR window sizes to show that our findings were not window length dependent. This ancillary validation yielded similar findings (details in Supplementary materials and Figs. S2, S3).

### Dynamic topological properties

Relative to controls, patients had lower coefficients of variation (CVs) of nodal efficiency and eigenvector centrality (EC) in the right medial prefrontal cortex (mPFC, part of DMN) and higher CVs of nodal EC in the left middle cingulate gyrus, left postcentral gyrus, right inferior temporal gyrus, bilateral paracentral lobules, and bilateral middle temporal gyrus, most of which are in SMN (Table 2). The CV of nodal EC in right mPFC in patients was negatively correlated with the total ratings (*r* = −0.29, *P* = 0.005) and general symptom ratings (*r* = −0.21, *P* = 0.048) from the positive and negative syndrome scale (PANSS) (Fig. 2b). There were no significant differences in temporal dynamics of global efficiency and cluster coefficient between groups. Most results were replicated using a larger (30 TR) sliding window (details in supplementary materials and Table S2).

**Table 2:** Case-control differences in coefficients of variation (CV) of nodal efficiency and eigenvector centrality.

Characteristics	Coordinates in MNI (*x*, *y*, *z*)	Network^a^	Uncorrected *P* value	FDR-corrected *P* value
*CV of nodal efficiency*				
Schizophrenia < controls				
right mPFC	9, 54, 3	DMN	<0.001	0.026
*CV of nodal eigenvector centrality*				
Schizophrenia < controls				
Right mPFC	9, 54, 3	DMN	0.002	0.049
Schizophrenia > controls				
Left middle temporal gyrus	−56, −13, −10	DMN	0.001	0.043
Right middle temporal gyrus	51, −29, −4	VAN	<0.001	0.005
Right paracentral lobule	3, −17, 58	SMN	<0.001	0.005
Right paracentral lobule	13, −33, 75	SMN	<0.001	0.022
Left paracentral lobule	−7, −33, 72	SMN	<0.001	0.005
Left postcentral gyrus	−23, −30, 72	SMN	<0.001	0.005
Left middle cingulate	0, −15, 47	SMN	<0.001	0.005
Right inferior temporal gyrus	52, −34, −27	Uncertain	<0.001	0.026

*MNI* Montreal Neurological Institute, *mPFC* medial prefrontal cortex, *DMN* default mode network, *SMN* sensorimotor network, *VAN* ventral attention network, *FDR* false discovery rate.

^a^Networks were defined by Power et al. atlas.

### Machine learning analysis

To verify the utility of dFC features for case identification at the individual level, *F* values were used to select features from temporal properties to include in the machine learning analysis, and a linear support vector classifier (SVC) was applied for individual classification. We gradually increased the number of features to find a balance between establishing an efficient and robust classifier while avoiding including too many features which could lead to overfitting (the number of features included varied from 1% to 100% of all temporal properties, with one percent increments). The SVC classifier showed 72.3% accuracy (area under the curve = 0.81) when 3% features were included, with 70.5% sensitivity and 74.0% specificity. The features selected every time across leave-one-out cross-validation (LOOCV) are shown in Table 3. The performance rate of classification was significantly higher than a randomly permuted group (*P* = 0.001). Almost all features included also showed significant between-group differences in the primary group comparisons (Table 3). The accuracy of the classification model is not high enough for clinical practice but is useful in showing that the modeled abnormalities together reflected commonly present brain features at the individual patient level.

**Table 3:** Features selected for classification model and the weights of these features in case-control classification.

Features	Coordinates in MNI (*x*, *y*, *z*)	Network^a^	Weights	Rank of absolute values of weights
*State characteristics*				
Mean dwell time of state 1^b^	–	–	0.86	11
*Nodal efficiency*				
Left fusiform gyrus	−47, −51, −21	Uncertain	3.80	2
Right angular gyrus	52, −59,36	DMN	−1.55	7
Right mPFC^b^	9, 54, 3	DMN	−1.12	9
*Nodal eigenvector centrality*				
Right middle temporal gyrus^b^	51, −29, −4	VAN	3.94	1
Left middle cingulate^b^	0, −15, 47	SMN	2.90	3
Right inferior temporal gyrus^b^	52, −34, −27	Uncertain	2.08	4
Right mPFC^b^	9, 54, 3	DMN	−2.01	5
Left paracentral lobule^b^	−7, −33, 72	SMN	1.64	6
Left middle temporal gyrus^b^	−56, −13, −10	DMN	1.53	8
Left fusiform gyrus	−47, −51, −21	Uncertain	−1.03	10
Left postcentral gyrus^b^	−23, −30, 72	SMN	0.86	12
Right paracentral lobule^b^	13, −33, 75	SMN	0.71	13
Right paracentral lobule^b^	3, −17, 58	SMN	0.12	14

^a^Networks were defined by Power et al. atlas.

^b^Indicates temporal properties that have previously exhibited between-group differences.

*MNI* Montreal Neurological Institute, *mPFC* medial prefrontal cortex, *DMN* default mode network, *SMN* sensorimotor network, *VAN* ventral attention network.

Lastly, in a subgroup of patients with follow-up data, the regression model with temporal properties contributing to the classification at baseline predicted a decline of PANSS total ratings over the course of treatment (*R*^2^ = 0.43, *P* < 0.01) at 6-week follow-up. Also, we observed a high accuracy (81.0%) of pretreatment data for differentiating short-term medication treatment responders/non-responders (details in supplementary materials and Tables S4–S9).

## Discussion

Our findings document the temporal and regional pattern of altered dynamic functional brain connectivity present in acutely ill, first-episode, never-treated patients with schizophrenia. Schizophrenia patients tended to remain in a weakly connected state (state 1) and spend less time in a more highly connected state (state 2). These findings suggest that previously observed static FC deficits in schizophrenia^27^ may reflect a reduced rate of shifting into the actively connected brain state as was observed in the present study.

Topological analyses found that schizophrenia patients demonstrated decreased functional dynamics in mPFC that were related to illness severity, and increased dynamics in temporal lobe and sensorimotor regions. Additionally, a machine learning analysis indicated that the observed pattern was commonly observed in individual schizophrenia patients. Importantly, these observations were present in acutely ill early-course patients prior to medication treatment, so that drug effects and course of illness effects did not confound the evaluation of illness-related brain features during acute psychosis.

### dFC states

The cluster analysis of dFC features identified two reoccurring dFC states representing discrete brain states of participants during scanning^28^. Compared with controls, patients with schizophrenia displayed more occurrences and dwelled longer in state 1 characterized by negative connectivities and relatively weak positive connectivities. The rate of transition between the states was also reduced in patients. These findings indicate that patients with schizophrenia demonstrate an atypical temporal distribution of functional brain states, with patients spending less time in a highly connected functional state and longer time in a weakly connected state.

Analysis of mean dwell time in states showed that patients with schizophrenia not only stayed in a weak state most of the time but also more quickly transitioned back away from the strongly connected state when it was engaged. Schizophrenia has been conceptualized as a disorder of decreased connectivity that is believed to be related to symptoms and cognitive impairment^27,29,30^. Our results indicate that the repeatedly observed reduction in average inter-region FC in schizophrenia^27^ is in part a result of more frequently staying in an “idling” state (state 1) rather than heightened connectivity in task-active networks^8^. Moreover, lower rates of switching into the more densely connected state, and faster exiting from that state when it was achieved, suggest resistance to shifting into more energy-demanding highly connected states required by task-active networks as previously reported^9^ that could contribute to the alterations of behavior and cognition associated with schizophrenia.

### Dynamic nodal topology

#### Decreased nodal dynamics in mPFC

The mPFC node of the DMN exhibited reduced time-varying nodal efficiency and EC. As a highly centralized hub region, the mPFC integrates information from abundant cortical and subcortical areas and assembles updated information transferred to multiple brain regions^31^. It plays an essential role in social behaviors, regulation of emotion, and cognitive processes such as decision-making^32^. Many previous studies have found that alterations in mPFC may lead to impairment of these functions in schizophrenia^33^. For example, weaker mPFC-amygdala coupling was associated with poorer social functioning and emotional dysfunction during the perception of emotional stimuli in schizophrenia^34,35^. Previous topological research in schizophrenia demonstrated that decreased centrality and efficiency of mPFC^16,36^ are caused by abnormal cortico-cortical fiber tract connectivity^37^ and FC^38,39^. Combined with the reduced fluctuation of topological properties found in our study, it appears that the centrality and efficiency of mPFC are kept at a low level and may not be able to change flexibly in schizophrenia. This hub region of DMN is functionally relatively locked in and less responsive to input and then less integrated into the broader brain organization because of the sustained low level of centrality and reduced coupling with other brain regions. This pathology could contribute to the dysfunction of overall brain topological structure with the brain not readily shifting out of the DMN into task-active states required by current contextual demands. Importantly, our exploratory correlational analyses of dynamic topology and symptoms (Fig. 2b) indicated that more severe symptoms of psychosis were associated with greater abnormalities of the dynamic topology of mPFC.

The disrupted connectivity between mPFC with other regions has been related to reduced coherent bursting of neural signaling in mPFC, which might be explained by N-methyl-D-aspartic acid hypofunction or altered dopaminergic signaling that are hallmarks of schizophrenia neurobiology^31^. Our finding of a correlation between dynamic mPFC nodal centrality and psychotic symptom severity before treatment highlight the clinical importance of dynamic functional alterations in mPFC in relation to whole brain topological organization.

#### Increased nodal dynamics in temporal and sensorimotor areas

We also identified nodes whose dynamic centrality was increased relative to HCs, including middle and inferior temporal gyri and sensorimotor cortex. The temporal lobes are important for auditory and linguistic processing and multimodal sensory integration^25,40^. The observations of increased dynamic connectivity in temporal lobes may represent a loss of functional controllability in these regions^14,41–43^. The increased dynamic nodal centrality in the temporal lobe, especially in auditory areas, may be due to internal and external sounds competing for attention (or being confused) during auditory processing^44^, a factor that could contribute to the auditory hallucinations that are common in schizophrenia. The lack of stability of graph metrics in the inferior temporal gyrus may lead to disorganized thinking and inferences about external events^45^. Dynamic centrality in temporal lobes carried a non-negligible weight in distinguishing first-episode schizophrenia patients from healthy individuals in our classification model, indicating that altered functional dynamics in the temporal lobe may commonly play an important role in the pathophysiology of acute illness in schizophrenia patients.

Other nodes with increased dynamic metrics belonged to the SMN and are involved in a variety of complex sensorimotor processing, features well established as being abnormal in schizophrenia^46,47^. The middle cingulate cortex is a central hub for motor planning and action monitoring systems^48^. Paracentral lobules and postcentral gyrus process sensations from different parts of the body and integrate sensorimotor responses in the planning of behavior^49^. These regions are also critical for emotional response systems^50,51^, including value scaling and utilizing action outcomes in behavioral planning^48^. Moderate levels of dynamic fluctuations are needed for optimizing context-relevant network activity, and for maintaining information processing with minimal metabolic expenditure^52^. Excessive fluctuations in brain nodes can lead to less efficient information transmission and aberrant behavior, and a failure in maintaining optimal metabolic costs^14,42^. Moreover, increased dynamic shifts may result in decreased nodal centrality in affected brain regions^38,53^, with adverse effects on information processing and behavior as seen in schizophrenia.

### Secondary analyses

The machine learning analysis focusing on the individual level yielded findings consistent with group average findings. These findings support the view that abnormal dynamic state characteristics and topology of these brain regions are likely to reflect core pathophysiological mechanisms at the individual level^12,19^. These findings support the view that disrupted dynamic functional integration of brain systems represents a common neurophysiological disturbance in schizophrenia. In addition, these dynamic properties have also been used to attempt to predict treatment outcomes and show potential in the available subset. The analysis of the follow-up study revealed that neurophysiological disturbances in brain system organization evident before treatment were clinically relevant in predicting treatment outcomes.

### Limitations

There are certain limitations to our study. Firstly, we used the sliding-window approach for evaluating changes in dFC. This is a widely used approach, but the best way to capture dynamic fluctuations remains controversial^54^. Using wavelet decompositions or dynamic conditional correlations instead of dFC might provide future advances in this field^53^. Secondly, the resting-state fMRI (rs-fMRI) scanning length in our study (400 s) was not long. A longer scan with a higher temporal resolution^55^ might provide a more refined assessment of brain functional dynamics in schizophrenia. There have been some dFC schizophrenia studies with a slightly longer scan time, which have generally indicated that schizophrenia is characterized by increased temporal variability in sensory and perceptual systems^14^ and decreased variability in default mode network and frontal-parietal network^11^. Thirdly, advances in dynamic modeling methods could improve single-time point resolution, such as instantaneous phase synchrony^56,57^ and multiplication of temporal derivatives^58^. Fourth, rs-fMRI data was acquired with a resolution of 3.75 × 3.75 × 5 mm^3^, and then resampled to a resolution of 3 × 3 × 3 mm^3^ before dynamic analysis. While providing isotropic voxels for analysis, this approach may have yielded higher estimates of within-region in the *z*-axis. Fifth, our statistical analysis suggested that the FC matrices were clustered into two categories and no other clusters (types of brain states) were identified. It is possible that unidentified or rare states may exist without the robustness to stand out as a discrete categorical type of brain functional state. It may also suggest a general consistency of brain state features across task-active networks in a rest state. Sixth, the classification model, while conducted for exploratory purposes of evaluating effects at the individual subject level, lacks validation in an external dataset. Seventh, from a clinical perspective, our findings are informative about acute psychosis at illness onset and provide preliminary evidence about relevance for predicting clinical changes after treatment, but the clinical relevance or nature of these findings later in the illness course, and their consistency across acute and stable clinical phases of illness, remain to be established. Lastly, while our exploratory follow-up study was informative, it was in a relatively small proportion of the original sample and thus our findings in this sample need to be considered with caution. Replication of the finding in a controlled clinical trial is needed to validate and extend information about the clinical relevance of our findings seen during the acute phase of illness.

## Conclusion

We demonstrated altered dFC in acutely ill, first-episode, never-treated patients with schizophrenia, characterized by spending more time in a state with more negative connectivities and relatively weak positive connectivities. In terms of topological properties, patients showed increased functional dynamics in the temporal lobe and sensorimotor regions and decreased dynamics in mPFC which was correlated with symptom severity. These altered dynamic properties were important contributors to distinguishing individual schizophrenia patients from controls. Our findings demonstrate the importance of evaluating dynamic aspects of brain connectivity to better understand the neurophysiological deficits associated with schizophrenia.

## Methods

### Participants

We recruited 102 acutely ill, first-episode, never-treated schizophrenia patients at West China Hospital of Sichuan University. Diagnoses were confirmed using the Structured Clinical Interview for DSM-IV Axis I Disorders (SCID) by two experienced psychiatrists. Clinical symptom severity was assessed with the PANSS. One hundred and three HCs were recruited from local communities by poster advertisement. The SCID interview (Nonpatient Edition) was used to rule out psychiatric illness in HCs. All HCs reported no known history of psychiatric illness in first-degree relatives. Exclusion criteria for all participants were as follows: (1) age less than 18 or more than 50; (2) left-handedness; (3) history of substance abuse or substance dependence; (4) presence of any other medical or neurological illness; (5) history of cerebral trauma or surgery, seizure, mental retardation, or brain imaging evidence of morphological anomalies; (6) current pregnancy or breastfeeding; and (7) MRI contraindications such as claustrophobia and metal or electronic implants. The study was approved by the local research ethics committee of West China Hospital of Sichuan University, and written informed consent was obtained from all participants.

### MRI data acquisition and preprocessing

All participants underwent rs-fMRI and high-resolution T1-weighted MRI using a 3.0 T MRI scanner (General Electric, Boston, USA) with an 8-channel phased-array head coil. MR image data preprocessing was carried out using the toolbox for Data Processing & Analysis of Brain Imaging (rfmri.org/DPABI, version 5.1) for removing the first ten volumes, slice-timing correction, head motion correction, nuisance signal regression, spatial normalization, smoothing, and filtering. Participants with a maximum head displacement of more than 1.5 mm or a maximum rotation greater than 1.5° were excluded from the analysis (excluding seven patients and three HCs). Ultimately, 95 antipsychotic-naive first-episode schizophrenia and 100 HCs were included in further analysis without significant between-group differences in age, sex, or years of education (Table 1). Details of the scanning parameters and rs-fMRI data preprocessing are provided in supplementary materials.

### Establishing dFC matrices

We used DynamicBC toolbox to generate dFC matrices (restfmri.net/forum/DynamicBC, version 2.2). The atlas of Power et al.^59^ delineates 264 cortical and subcortical brain nodes, with 10 mm diameter spheres centered in each of them that can be used for FC analysis. The 264 spheres were divided into 13 functional networks^59^: DMN, VN, FPN, CON, AN, SN, sensorimotor hand network (SMN_hand), sensorimotor mouth network (SMN_mouth), dorsal attention network (DAN), VAN, memory retrieval network (MN), subcortical network, and cerebellar network. The mean time series of blood oxygen level-dependent signals were extracted from voxels in each spherical brain node for analysis. FC between these defined 264 regions was calculated via Pearson’s correlation to generate FC matrices. A sliding-window approach was used to explore the time-varying changes of FC between each pair of brain nodes. All the time series were segmented into overlapping time windows and slid with a step of one time of repetition (TR) along with the time series. We chose a 22TR window (44 s) because previous studies have suggested that windows of 30 s to 60 s can successfully capture patterns of resting-state fluctuations of connectivity^60^ and such window sizes have typically been used in dFC studies^43,61^. During the whole scanning period, there are 169 dFC matrices per person when there were 190 brain scanning volumes and 22TR window sizes and the step was 1TR (details in Supplementary methods).

### dFC state analysis

To identify discrete reoccurring patterns of dFC states across subjects, we performed *k*-means clustering with Scikit-Learn (version 0.23.1) on Python (version 3.8.3) on a series of 264 × 264 FC matrices for all participants. The similarity between each windowed FC matrix was estimated using the Euclidean distance. To identify the common and robust discrete categorical data organization, the *k* value was varied from two to ten to search the number of such groups of events in the data before clustering. We used the “elbow criteria” to find the inflection of the sum of the squared errors and applied the maximum silhouette coefficient together to determine the optimal *k* value. Both methods which aim to identify the number of types of overall data organization (here, types of brain states) indicated the best *k* to be two in the present study (Fig. S1), indicating that there were two types of functional brain states in the data. In primary analyses, we used the cluster centroids of all participants to represent the two reoccurring dFC states (Fig. 1a). For visualization of dFC states in the two groups respectively, we also calculated the group-specific cluster centroids (Fig. 1b). To examine temporal properties of the two dFC states, we assessed three different state characteristics: (1) fractional time, the proportion of time window belonging to each state; (2) mean dwell time, the average length of consecutive time windows spent in each state; and (3) number of transitions, the number of switches between states over time.

### Dynamic topological analysis

The analysis of dynamic functional topology can characterize how all brain regions work together and delineate anatomic and functional sources of aberrant dFC^14,36^. To avoid a different number of edges caused by weak spurious FCs and reduce the computational complexity in the assessment of topological architecture, the top K% (*K* = 5–20 with an interval of 5) strength of the positive FCs for each 264 × 264 FC matrix were kept to obtain a series of sparse weighted matrices. To represent global and regional characteristics of topological networks, global efficiency, clustering coefficient, nodal efficiency, and nodal EC were computed for each sliding window for each person at each sparsity using the Brain Connectivity Toolbox (nitrc.org/projects/bct, version 2019-03-03). A detailed interpretation of these topological characteristics is presented in Table S1. The area under the curve (AUC) measures across the range of sparsity thresholds were obtained to ensure the robustness of the four topological characters. The CV of the AUC of all topological metrics was calculated across all time windows to characterize temporal variability for all subjects. High CVs reflect high fluctuation rates of topological metrics, and low CV values reflect more stable states. To assess the consistency and validity of the dynamic analysis at different window sizes, we used another sliding window parameter (30TR window width and 2TR step length) to repeat the above analyses and validate the results. The results of findings from the longer window did not meaningfully differ from those of the primary analysis. The results reported in the main text are under 22TR window sizes, and the results of 30TR were reported in the supplementary materials.

### Statistical analysis

All neuroimaging statistical analysis was performed in MATLAB R2017b. Permutation testing iterated 10,000 times was conducted to test for group differences due to the non-normal distribution of state characteristics and dynamic topological metrics (details in supplementary methods). Significance was set at *P* < 0.05 with FDR correction for multiple comparisons. In patients, we performed Spearman partial correlations to explore relations of dynamic properties (state characteristics and CVs of topological metrics showing intergroup differences) with acute illness severity (PANSS ratings), treating demographic measures (age, sex, and education years) as covariates. Nominal significance was set at *P* < 0.05 for these correlation analyses conducted for heuristic and descriptive purposes.

### Classification based on dynamic properties

We next identified the brain features that most robustly and consistently distinguished individual schizophrenia patients from HCs using a linear SVC. The feature selection by F score was performed instead of directly using the previous features with significant between-group differences to avoid data leakage from the training set to the test set and reduce the risk of overfitting. After that, SVC was trained by the selected features. LOOCV was performed to evaluate model generalization. Permutation testing was conducted to test the performance of the classifier.

In an additional exploratory analysis with a subsample of 21 patients followed clinically after 6 weeks of antipsychotic treatment (Table S3), we determined whether the dynamic properties contributing to the classification model could predict clinical treatment response with support vector regression. The prediction of short-term treatment response procedures is described in detail in supplementary materials.

## Supplementary information

supplemental material

## Data Availability

The custom code that supports the findings of this study is available (github.com/youwanfang/Dynamic-Properties). The clinical data are not publicly available because of ethical restrictions that protect patients’ privacy and consent. Other data that support the findings of this study are available from the corresponding author L.F. upon reasonable request.
