# Supplementary material for: Impaired dynamic functional brain properties and their relationship to symptoms in never treated first-episode patients with schizophrenia
Source: Schizophrenia (Heidelb). 2022 Oct 29;8(1):90. doi: 10.1038/s41537-022-00299-9 (PMC9617869; doi:10.1038/s41537-022-00299-9)
Supplement: Supplementary file 1 — supplemental material [file 41537_2022_299_MOESM1_ESM.docx]

**Supplementary Materials**

[**Supplementary Methods** 3](#_Toc115265084)

[**Data acquisition and preprocessing** 3](#_Toc115265085)

[**Number of dynamic functional connectivity (dFC) matrices** 3](#_Toc115265086)

[**Interpretation of topological characteristics** 4](#_Toc115265087)

[**Permutation test** 4](#_Toc115265088)

[**Classification based on dynamic properties** 5](#_Toc115265089)

[**Predict short-term treatment response** 5](#_Toc115265090)

[**Supplementary Results** 6](#_Toc115265091)

[**Validation of abnormalities of dFC state and dynamic topological properties** 6](#_Toc115265092)

[**Performance of predicting treatment effect** 7](#_Toc115265093)

[**Table S1.** Interpretations and mathematical formulae of topological properties used in the present study. 9](#_Toc115265094)

[**Table S2.** The between-group differences in coefficients of variation (CV) of nodal efficiency and eigenvector centrality for validation. 10](#_Toc115265095)

[**Table S3.** Symptom severity details of the subsample with a six-week follow-up. 11](#_Toc115265096)

[**Table S4.** The predicted drop of PANSS total ratings and treatment response at six-week follow-up in schizophrenia (SZ) patients. 12](#_Toc115265097)

[**Table S5.** The predicted drop of PANSS positive symptom ratings at six-week follow-up in patients. 13](#_Toc115265098)

[**Table S6.** The predicted drop of PANSS general symptom ratings at six-week follow-up in patients. 14](#_Toc115265099)

[**Table S7.** The weights of features in the prediction of PANSS total ratings at six-week follow-up in patients. 15](#_Toc115265100)

[**Table S8.** The weights of features in the prediction of PANSS positive symptom ratings at six-week follow-up in patients. 16](#_Toc115265101)

[**Table S9.** The weights of features in the prediction of PANSS general symptom ratings at six-week follow-up in patients. 17](#_Toc115265102)

[**Figure S1.** Estimating the optimal number of k value. The ordinate of the "elbow criteria" is the sum of the squared errors (SSE). The relationship between SSE and k is in the shape of an “elbow”, and the k value at inflection point is the actual number of clusters of the data. Here, the optimal k value is two (a). The "silhouette coefficient" reflects the sample distance. The k value at the largest average silhouette coefficient is the optimal number of clusters, which also indicates k value is two (b). 18](#_Toc115265103)

[**Figure S2.** Cluster centroids of reoccurring dynamic functional connectivity patterns with 30TR window and 2TR step length for validation. Cluster centroids for each state across all participants (a).Cluster centroids for each state for each group (b). The color bar shows the strength of the functional connectivity between two brain nodes. For the abbreviations of brain networks, see Figure 1. 19](#_Toc115265104)

[**Figure S3.** Comparison of state characteristics in patients and controls for validation. Patients with schizophrenia (SZs) showed altered fractional time in two states (a), decreased mean dwell time of state 2 (b), and decreased transitions between states (c) than healthy controls (HCs). * indicates false discovery rate corrected *P* value < 0.05. 20](#_Toc115265105)

[**References** 21](#_Toc115265106)

**Supplementary Methods**

**Data acquisition and preprocessing**

The resting-state functional MRI (rs-fMRI) data were obtained via an echo-planar imaging sequence with the following parameters: time of repetition/echo (TR/TE) = 2000/30 ms; flip angle = 90°; slice thickness = 5 mm (no slice gap), field of view (FOV) = 240 × 240 mm^2^, matrix size = 64 × 64, voxel size = 3.75 × 3.75 × 5 mm^3^. Each brain volume comprised 30 axial slices, and each functional session contained 200 volumes. High-resolution T1-weighted volumetric 3-dimensional images were obtained using a spoiled gradient recall sequence (TR/TE = 8.5/3.4 ms; flip angle = 12°; slice thickness = 1 mm; FOV = 240 × 240 mm^2^; matrix size = 256 × 256; number of axial slices = 156). Participants were instructed to keep their heads still and relax with eyes closed without systemic thought or falling asleep during scanning. Foam padding and earplugs were used to reduce head motion and scanner noise.

The first ten volumes of rs-fMRI data were removed to reduce equilibration effects, leaving a total of 190 volumes for further analysis. The remaining functional images underwent slice-timing correction and were realigned to reduce displacement between volumes. To control for head motion and physiological noises, the Friston-24 head motion parameters, linear trend, and signals from the white matter and cerebrospinal fluid were regressed out. Subsequently, the rs-fMRI data were warped to the Montreal Neurological Institute template and were resampled to 3 × 3 × 3 mm^3^ resolution. Then the normalized rs-fMRI data were spatially smoothed using a 4 mm full width at half maximum Gaussian kernel. Finally, the normalized data were temporally bandpass filtered (0.01 to 0.1 Hz). Regarding head motion during scanning, the mean displacement (0.11 ± 0.08 for patients, 0.11 ± 0.06 for controls, *t* = 0.171, *P* = 0.864) and mean rotation (0.10 ± 0.07 for patients, 0.12 ± 0.08 for controls, *t* = 1.694, *P* = 0.092) showed no significant differences between patients and controls.

**Number of dynamic functional connectivity (dFC) matrices**

The number of dFC matrices for each person is the number of windows for each blood oxygen level dependent signal. For the brain volume *V* and *W* window size with *S* step length, the number of windows *N* can be computed as:

*N* = $\frac{V-W}{S} +$1

In our study, there are 190 brain volumes (10 volumes have been removed before analysis). Then, there are 169 matrices under 22TR window width and 81 matrices under 30TR window width.

**Interpretation of topological characteristics**

Global efficiency is the inverse average shortest path length of each node which measures how efficiently information is transferred at the global level^1^. Cluster coefficient is a fraction of the neighboring nodes also being neighbors of each other, which measures the extent to which the network can be segregated^2^. Nodal efficiency reflects the efficiency of each node and the resilience of the network to local failures^3^. Nodal eigenvector centrality (EC) was used to measure the centrality by combining the strength of the connection between nodes and neighboring nodes and the importance of the neighboring nodes themselves^4^ (Table S1).

**Permutation test**

The original distribution of the data (state characteristics and dynamic topological properties) was nonnormal, so permutation test was performed to compare patients and controls. The dynamic properties from the two groups were put together and shuffled, and then redistributed into two groups as the original sample sizes. Then, we calculated the difference between the means of the two resampled groups. This step was repeated 10,000 times to generate a series difference of means, which constitutes the null distributed data. The *P* value was estimated as the percentage of null data that exceeded the difference between the means on the original data.

**Classification based on dynamic properties**

We examined whether dynamic properties (state characteristics and dynamic topological properties) have the potential ability to distinguish patients with schizophrenia (SZs) from healthy controls (HCs) with a linear support vector classifier (SVC). This part was implemented in MATLAB R2017b and SVC algorithm is available from LIBSVM (csie.ntu.edu.tw/~cjlin/libsvm). There were 195 samples (95 patients and 100 HCs) along with 534 features (4 state characteristics, coefficients of variation [CV] of global efficiency, CV of clustering coefficient, 264 CVs of nodal efficiency, and 264 CVs of nodal EC). The leave-one-out cross-validation (LOOCV) was repeated 195 times to evaluate model generalization ability.

We calculated F score which is a ratio of the between-group variation divided by the within-group variation to select features. The larger F score is, the more discriminative the feature is. The feature selection among the 534 features was performed instead of directly using the previous features with significant group differences, to avoid data leakage from the training set to the test set and reduce the risk of overfitting. After that, SVC was trained by these selected features in the training set and investigated the performance in the test sample. In addition, permutation test was conducted 1000 times to test the significance. We observed the performance and significance of the classification. The features, which were selected every time across LOOCV contributing to distinguishing patients and controls and simultaneously displayed between-group differences in previous univariate statistical analyses, were concerned as much meaningful imaging markers for further discussion in the present study.

**Predict short-term treatment response**

We performed exploratory research about whether the brain dynamic properties of patients before the medical treatment could predict the short-term clinical treatment response. There were 27 patients with schizophrenia instructed to take medication regularly based on their prescriptions after the baseline clinical and rs-fMR imaging assessment. Drug and dose choices were made independently by treating psychiatric physicians according to the clinical preference of patients. Except for medication, none of the patients received other types of treatment across the entire follow-up period. At the six-week follow-up, the treatment response was assessed by the severity of symptoms with Positive and Negative Syndrome Scale (PANSS). In the end, 21 patients followed the medication regimens and cooperated to complete the follow-up PANSS test (Table S3), who were included in further analysis. The 21 patients were treated with risperidone (n = 14), quetiapine (n = 5), clozapine (n = 5), olanzapine (n = 3), sulpiride (n = 2) and aripiprazole (n = 1).

We applied linear support vector regression (SVR) to predict drop of PANSS total ratings, positive symptoms, negative symptoms, and general symptoms after treatment, respectively. Brain temporal properties robustly contributing to classification at baseline (as illustrated in Table 3) together with sex and age of patients were used to train models. This prognostic prediction analysis was implemented in MATLAB R2017b and the SVR algorithm is available from LIBSVM (csie.ntu.edu.tw/~cjlin/libsvm). The LOOCV was applied to ensure the robustness of the prediction results. The performance of SVR models was evaluated by the coefficient of determination (*R*^2^) and significance of 1000 permutation tests. Meanwhile, the treatment effect was considered responsive if the reduction ratio of PANSS total ratings was more than 30%. The PANSS total ratings reduction ratio is defined as the relative change in PANSS total ratings from baseline to six-week follow-up, calculated as (PANSS_baseline_ - PANSS_followup_) / (PANSS_baseline_ - 30).

**Supplementary Results**

**Validation of abnormalities of dFC state and** **dynamic topological properties**

In this part, dynamic state characteristics and dynamic topological properties were calculated with 30TR window wide and 2TR steps. For the validation of dynamic state analysis, we found that the fractional time of state 1 was significantly higher in SZs than HCs (SZs 75.5 ± 28.1%, HCs 67.1 ± 30.7%, FDR-*P* = 0.046). Accordingly, patients spent less time in state 2 compared with controls (SZs 24.5 ± 28.1%, HC 32.9 ± 30.7%, FDR-*P* = 0.046). The mean dwell time in hypoconnected state 1 showed no group differences (SZs 40.4 ± 30.2, HCs 29.3 ± 24.9, uncorrected *P* = 0.118), and patients demonstrated reduced mean dwell time in hyperconnected state 2 (SZs 9.2 ± 12.1, HCs 11.4 ± 12.9, FDR-*P* = 0.009). The number of transitions between states decreased in SZs than HCs (SZs 3.0 ± 3.1, HCs 3.7 ± 2.8, FDR-*P* = 0.052) (Figures S2 and S3). No significant correlations between these state characteristics and clinical information were observed in patients.

The CV of nodal efficiency in left fusiform gyrus was higher and that in right mPFC was lower in SZs than HCs. Besides, patients had higher CVs of nodal EC in left middle cingulate gyrus, left postcentral gyrus, left superior temporal gyrus, right inferior temporal gyrus bilateral paracentral lobules, and bilateral middle temporal gyrus compared with controls (Table S2). In addition, there was a trend that patients had a lower CV of EC in right mPFC (uncorrected, *P* = 0.029) relative to controls. The CV of nodal EC in right mPFC in patients was negatively correlated with the total ratings (*r* = -0.239, *P* = 0.022) of PANSS. There were no significant differences in terms of temporal dynamics of global efficiency and cluster coefficient between groups.

**Performance of predicting treatment effect**

The SVR models with temporal properties could predict the drop of PANSS total ratings (*R*^2^ = 0.43, *P* < 0.01), positive symptom ratings (*R*^2^ = 0.35, *P* = 0.003), and general symptom ratings (*R*^2^ = 0.32, *P* = 0.001), but not negative symptoms. According to the definition of treatment response, 15 SZs as responders had a decrease in PANSS total ratings of a minimum of 30%, and the other six patients were non-responders (Table S4). The regression model about the drop ratings of PANSS could predict response and non-response with an accuracy of 81.0% (93.3% accuracy in responders and 50.0% accuracy in non-responders). The detailed prediction results were shown in Tables S4-S9.

**Table S1.** Interpretations and mathematical formulae of topological properties used in the present study.

| Measures | Interpretations and mathematical formulae |
| --- | --- |
| Global efficiency (*E_glob_*) | The global efficiency is the average of inverse shortest path length and measures the ability of parallel information transmission over the network. For the global network *G* with *N* nodes and *K* edges, the global efficiency can be computed as:  *E_glob_(G)*=$\frac{1}{N(N-1)}\sum_{i\neq jG} \frac{1}{d_{ij}}$  Where *d_ij_* is the shortest path length between node *i* and node *j* in *G*. |
| Clustering coefficient (*C*) | The clustering coefficient (*C*) for whole brain network is the average of the clustering coefficients over all nodes in the global network (*G*). The clustering coefficient of a node *i* (*C_i_*) is defined as the “intensity” (geometric mean) of triangles, which measures the extent to which neighbors of a node also connect to each other. The overall weighted clustering coefficient (*C*) is defined as follows:  $C=\frac{1}{N}\sum_{i\in G} C_{i}$ |
| Nodal efficiency (*e*) | The nodal efficiency for a given node measures the efficiency of parallel information transfer of that node in the network and the resilience of the temporal network to local failures. The nodal efficiency of node *i* is computed as:  *e_i_* _=_ $\sum_{m\neq i\neq nG} \frac{\sigma_{mn}(i)}{\sigma_{mn}}$  where σ*_mn_* is the total number of shortest paths from node *m* to node *n* and σ*_mn_* (*i*) is the number of shortest paths from node *m* to node *n* that pass through the node *i*. |
| Nodal eigenvector centrality (*v*) | The nodal eigenvector centrality measures the centrality combining the strength of the connection between nodes and neighboring nodes and the importance of the neighboring nodes themselves. In a network *G* with *N* nodes, the eigenvector centrality *v_i_* of node *i* is defined as:  *v_i_=*$\mu\sum_{jG} a_{ij}v_{j}$  where *a_ij_* is the *i* th row and *j* th column element of the adjacency matrix *A* for *N* nodes. The *x_i_* is proportional to the sum of similarity scores of all connected nodes when the *μ* = 1/*λ*, where the *λ* is the largest eigenvalue. |

**Table S2.** The between-group differences in coefficients of variation (CV) of nodal efficiency and eigenvector centrality for validation.

| Regions | Coordinates in MNI (x, y, z) | Network# | Uncorrected *P* value | FDR corrected *P* value |
| --- | --- | --- | --- | --- |
| **CV of nodal efficiency** |  |  |  |  |
| Schizophrenia > controls |  |  |  |  |
| left fusiform gyrus | -47, -51, -21 | uncertain | < 0.001 | 0.013 |
| Schizophrenia < controls | |  |  |  |
| right mPFC | 9, 54, 3 | DMN | < 0.001 | 0.013 |
| **CV of nodal eigenvector centrality** | |  |  |  |
| Schizophrenia > controls | |  |  |  |
| left middle temporal gyrus | -56, -13, -10 | DMN | 0.001 | 0.041 |
| right middle temporal gyrus | 51, -29, -4 | VAN | <0.001 | 0.007 |
| right paracentral lobule | 3, -17, 58 | SMN | <0.001 | 0.007 |
| right paracentral lobule | 13, -33, 75 | SMN | <0.001 | 0.030 |
| left paracentral lobule | -7, -33, 72 | SMN | <0.001 | 0.030 |
| left postcentral gyrus | -23, -30, 72 | SMN | <0.001 | 0.007 |
| left middle cingulate | 0, -15, 47 | SMN | 0.001 | 0.041 |
| right inferior temporal gyrus | 52, -34, -27 | uncertain | <0.001 | 0.007 |
| left superior temporal gyrus | -60, -25, 14 | AN | <0.001 | 0.030 |

#, networks were defined by Power et al. atlas.

Abbreviations: MNI, Montreal Neurological Institute; mPFC, medial prefrontal cortex; DMN, default mode network; SMN, sensorimotor network; VAN, ventral attention network; AN, auditory network; FDR, false discovery rate.

**Table S3.** Symptom severity details of the subsample with a six-week follow-up.

|  | Baseline  (N = 21) | After treatment  (N = 21) | *t* value* | *P* value |
| --- | --- | --- | --- | --- |
| PANSS total ratings | 98.6±11.4 | 66.1±17.8 | 10.567 | <0.001 |
| PANSS positive symptom ratings | 28.4±5.1 | 14.6±4.5 | 10.244 | <0.001 |
| PANSS negative symptom ratings | 19.5±6.8 | 16.4±6.3 | 2.837 | 0.01 |
| PANSS general symptom ratings | 50.6±7.4 | 35.1±9.5 | 9.002 | <0.001 |

*, paired *t*-test.

**Table S4.** The predicted drop of PANSS total ratings and treatment response at six-week follow-up in schizophrenia (SZ) patients.

| Patient number | Actual PANSS total ratings | |  | Drop of PANSS total ratings after treatment | |  | Treatment response | |
| --- | --- | --- | --- | --- | --- | --- | --- | --- |
|  | Baseline | After treatment |  | Actual drop | Predicted drop |  | Actual response | Predicted response |
| SZ-01 | 87 | 44 |  | 43 | 35.5 |  | Responder | Responder |
| SZ-02 | 96 | 39 |  | 57 | 37.7 |  | Responder | Responder |
| SZ-03 | 87 | 69 |  | 18 | 21.7 |  | Responder | Responder |
| SZ-04 | 92 | 55 |  | 37 | 26.8 |  | Responder | Responder |
| SZ-05* | 117 | 99 |  | 18 | 43.8 |  | Non-responder | Responder |
| SZ-06 | 83 | 49 |  | 34 | 35.5 |  | Responder | Responder |
| SZ-07 | 104 | 68 |  | 36 | 42.2 |  | Responder | Responder |
| SZ-08* | 108 | 85 |  | 23 | 29.7 |  | Non-responder | Responder |
| SZ-09 | 105 | 89 |  | 16 | 16.8 |  | Non-responder | Non-responder |
| SZ-10 | 102 | 50 |  | 52 | 41.9 |  | Responder | Responder |
| SZ-11* | 81 | 61 |  | 20 | 9.0 |  | Responder | Non-responder |
| SZ-12 | 113 | 103 |  | 10 | 22.6 |  | Non-responder | Non-responder |
| SZ-13 | 90 | 66 |  | 24 | 28.7 |  | Responder | Responder |
| SZ-14 | 93 | 57 |  | 36 | 36.6 |  | Responder | Responder |
| SZ-15 | 114 | 90 |  | 24 | 23.0 |  | Non-responder | Non-responder |
| SZ-16 | 112 | 63 |  | 49 | 38.4 |  | Responder | Responder |
| SZ-17 | 108 | 63 |  | 45 | 44.6 |  | Responder | Responder |
| SZ-18 | 90 | 52 |  | 38 | 40.4 |  | Responder | Responder |
| SZ-19 | 95 | 56 |  | 39 | 39.2 |  | Responder | Responder |
| SZ-20* | 84 | 72 |  | 12 | 29.0 |  | Non-responder | Responder |
| SZ-21 | 109 | 59 |  | 50 | 38.8 |  | Responder | Responder |

* indicates the predictive treatment response for these few people was wrong.

**Table S5.** The predicted drop of PANSS positive symptom ratings at six-week follow-up in patients.

| Patient number | Actual PANSS positive symptom ratings | |  | Drop of PANSS positive symptom ratings after treatment | |
| --- | --- | --- | --- | --- | --- |
|  | Baseline | After treatment |  | Actual drop | Predicted drop |
| SZ-01 | 26 | 9 |  | 17 | 17.5 |
| SZ-02 | 25 | 8 |  | 17 | 17.4 |
| SZ-03 | 25 | 16 |  | 9 | 7.0 |
| SZ-04 | 19 | 9 |  | 10 | 9.6 |
| SZ-05 | 27 | 21 |  | 6 | 15.9 |
| SZ-06 | 21 | 11 |  | 10 | 17.2 |
| SZ-07 | 33 | 13 |  | 20 | 15.0 |
| SZ-08 | 35 | 23 |  | 12 | 16.9 |
| SZ-09 | 24 | 17 |  | 7 | 10.2 |
| SZ-10 | 35 | 11 |  | 24 | 23.0 |
| SZ-11 | 25 | 17 |  | 8 | 9.3 |
| SZ-12 | 28 | 22 |  | 6 | 11.9 |
| SZ-13 | 33 | 18 |  | 15 | 12.0 |
| SZ-14 | 32 | 15 |  | 17 | 15.7 |
| SZ-15 | 24 | 12 |  | 12 | 10.7 |
| SZ-16 | 37 | 12 |  | 25 | 17.3 |
| SZ-17 | 34 | 11 |  | 23 | 16.9 |
| SZ-18 | 34 | 13 |  | 21 | 14.7 |
| SZ-19 | 27 | 11 |  | 16 | 18.2 |
| SZ-20 | 26 | 18 |  | 8 | 10.7 |
| SZ-21 | 27 | 19 |  | 8 | 17.8 |

**Table S6.** The predicted drop of PANSS general symptom ratings at six-week follow-up in patients.

| Patient number | Actual PANSS general symptom ratings | |  | Drop of PANSS general symptom ratings after treatment | |
| --- | --- | --- | --- | --- | --- |
|  | Baseline | After treatment |  | Actual drop | Predicted drop |
| SZ-01 | 47 | 24 |  | 23 | 24.2 |
| SZ-02 | 54 | 22 |  | 32 | 17.9 |
| SZ-03 | 45 | 35 |  | 10 | 11.9 |
| SZ-04 | 39 | 27 |  | 12 | 7.9 |
| SZ-05 | 62 | 53 |  | 9 | 26.1 |
| SZ-06 | 46 | 25 |  | 21 | 16.2 |
| SZ-07 | 56 | 39 |  | 17 | 22.7 |
| SZ-08 | 58 | 47 |  | 11 | 12.6 |
| SZ-09 | 60 | 51 |  | 9 | 4.0 |
| SZ-10 | 52 | 28 |  | 24 | 15.3 |
| SZ-11 | 41 | 33 |  | 8 | 1.9 |
| SZ-12 | 51 | 48 |  | 3 | 10.0 |
| SZ-13 | 43 | 31 |  | 12 | 16.6 |
| SZ-14 | 47 | 29 |  | 18 | 19.8 |
| SZ-15 | 58 | 51 |  | 7 | 10.9 |
| SZ-16 | 60 | 35 |  | 25 | 20.8 |
| SZ-17 | 56 | 34 |  | 22 | 18.6 |
| SZ-18 | 43 | 28 |  | 15 | 20.6 |
| SZ-19 | 48 | 34 |  | 14 | 19.6 |
| SZ-20 | 39 | 33 |  | 6 | 10.2 |
| SZ-21 | 58 | 31 |  | 27 | 22.2 |

**Table S7.** The weights of features in the prediction of PANSS total ratings at six-week follow-up in patients.

| Features | Coordinates in MNI (x, y, z) | Network# | Weights | Rank of absolute values of weights |
| --- | --- | --- | --- | --- |
| **Demographic** |  |  |  |  |
| sex | -- | -- | 15.37 | 1 |
| age | -- | -- | -3.15 | 7 |
| **State characteristics** |  |  |  |  |
| mean dwell time of state 1 | -- | -- | -2.27 | 10 |
| **Nodal efficiency** |  |  |  |  |
| left fusiform gyrus | -47, -51, -21 | uncertain | 6.44 | 5 |
| right angular gyrus | 52, -59,36 | DMN | 2.16 | 11 |
| right mPFC | 9, 54, 3 | DMN | 2.37 | 9 |
| **Nodal eigenvector centrality** | |  |  |  |
| right middle temporal gyrus | 51, -29, -4 | VAN | 7.02 | 4 |
| left middle cingulate | 0, -15, 47 | SMN | 0.74 | 13 |
| right inferior temporal gyrus | 52, -34, -27 | uncertain | 1.03 | 12 |
| right mPFC | 9, 54, 3 | DMN | -4.24 | 6 |
| left paracentral lobule | -7, -33, 72 | SMN | 2.62 | 8 |
| left middle temporal gyrus | -56, -13, -10 | DMN | 10.57 | 2 |
| left fusiform gyrus | -47, -51, -21 | uncertain | 9.88 | 3 |
| left postcentral gyrus | -23, -30, 72 | SMN | 0.56 | 14 |
| right paracentral lobule | 13, -33, 75 | SMN | -0.31 | 15 |
| right paracentral lobule | 3, -17, 58 | SMN | -0.06 | 16 |

#, networks were defined by Power et al. atlas.

Abbreviations: MNI, Montreal Neurological Institute; mPFC, medial prefrontal cortex; DMN, default mode network; SMN, sensorimotor network; VAN, ventral attention network.

**Table S8.** The weights of features in the prediction of PANSS positive symptom ratings at six-week follow-up in patients.

| Features | Coordinates in MNI (x, y, z) | Network# | Weights | Rank of absolute values of weights |
| --- | --- | --- | --- | --- |
| **Demographic** |  |  |  |  |
| sex | -- | -- | 5.20 | 2 |
| age | -- | -- | -2.06 | 4 |
| **State characteristics** |  |  |  |  |
| mean dwell time of state 1 | -- | -- | 0.41 | 13 |
| **Nodal efficiency** |  |  |  |  |
| left fusiform gyrus | -47, -51, -21 | uncertain | 0.48 | 11 |
| right angular gyrus | 52, -59,36 | DMN | 0.96 | 9 |
| right mPFC | 9, 54, 3 | DMN | 2.68 | 3 |
| **Nodal eigenvector centrality** | |  |  |  |
| right middle temporal gyrus | 51, -29, -4 | VAN | 1.95 | 5 |
| left middle cingulate | 0, -15, 47 | SMN | 0.01 | 16 |
| right inferior temporal gyrus | 52, -34, -27 | uncertain | -0.42 | 12 |
| right mPFC | 9, 54, 3 | DMN | -0.35 | 14 |
| left paracentral lobule | -7, -33, 72 | SMN | 0.91 | 10 |
| left middle temporal gyrus | -56, -13, -10 | DMN | -1.13 | 7 |
| left fusiform gyrus | -47, -51, -21 | uncertain | 6.06 | 1 |
| left postcentral gyrus | -23, -30, 72 | SMN | -1.26 | 6 |
| right paracentral lobule | 13, -33, 75 | SMN | -0.25 | 15 |
| right paracentral lobule | 3, -17, 58 | SMN | 1.02 | 8 |

#, networks were defined by Power et al. atlas.

Abbreviations: MNI, Montreal Neurological Institute; mPFC, medial prefrontal cortex; DMN, default mode network; SMN, sensorimotor network; VAN, ventral attention network.

**Table S9.** The weights of features in the prediction of PANSS general symptom ratings at six-week follow-up in patients.

| Features | Coordinates in MNI (x, y, z) | Network# | Weights | Rank of absolute values of weights |
| --- | --- | --- | --- | --- |
| **Demographic** |  |  |  |  |
| sex | -- | -- | 9.43 | 1 |
| age | -- | -- | 1.29 | 10 |
| **State characteristics** |  |  |  |  |
| mean dwell time of state 1 | -- | -- | -2.87 | 5 |
| **Nodal efficiency** |  |  |  |  |
| left fusiform gyrus | -47, -51, -21 | uncertain | 1.76 | 8 |
| right angular gyrus | 52, -59,36 | DMN | -1.95 | 7 |
| right mPFC | 9, 54, 3 | DMN | -0.38 | 13 |
| **Nodal eigenvector centrality** | |  |  |  |
| right middle temporal gyrus | 51, -29, -4 | VAN | 1.66 | 9 |
| left middle cingulate | 0, -15, 47 | SMN | 0.29 | 15 |
| right inferior temporal gyrus | 52, -34, -27 | uncertain | -0.93 | 11 |
| right mPFC | 9, 54, 3 | DMN | -3.22 | 4 |
| left paracentral lobule | -7, -33, 72 | SMN | 0.32 | 14 |
| left middle temporal gyrus | -56, -13, -10 | DMN | 4.32 | 3 |
| left fusiform gyrus | -47, -51, -21 | uncertain | 6.60 | 2 |
| left postcentral gyrus | -23, -30, 72 | SMN | -2.56 | 6 |
| right paracentral lobule | 13, -33, 75 | SMN | -0.17 | 16 |
| right paracentral lobule | 3, -17, 58 | SMN | 0.61 | 12 |

#, networks were defined by Power et al. atlas.

Abbreviations: MNI, Montreal Neurological Institute; mPFC, medial prefrontal cortex; DMN, default mode network; SMN, sensorimotor network; VAN, ventral attention network.

**Figure S1.** Estimating the optimal number of k value. The ordinate of the "elbow criteria" is the sum of the squared errors (SSE). The relationship between SSE and k is in the shape of an “elbow”, and the k value at inflection point is the actual number of clusters of the data. Here, the optimal k value is two (a). The "silhouette coefficient" reflects the sample distance. The k value at the largest average silhouette coefficient is the optimal number of clusters, which also indicates k value is two (b).


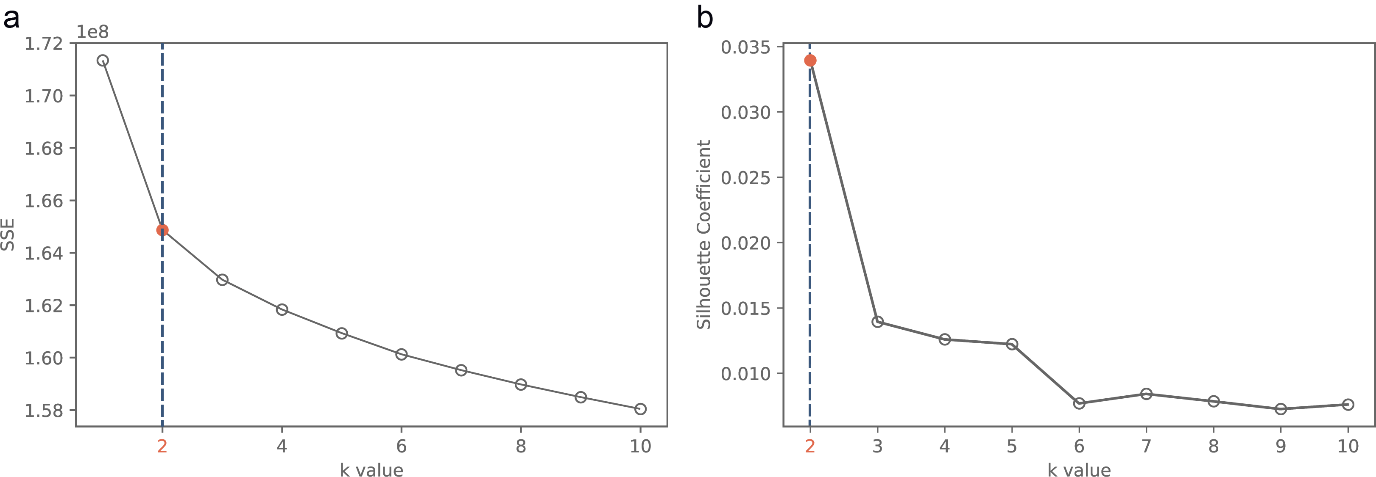


**Figure S2.** Cluster centroids of reoccurring dynamic functional connectivity patterns with 30TR window and 2TR step length for validation. **
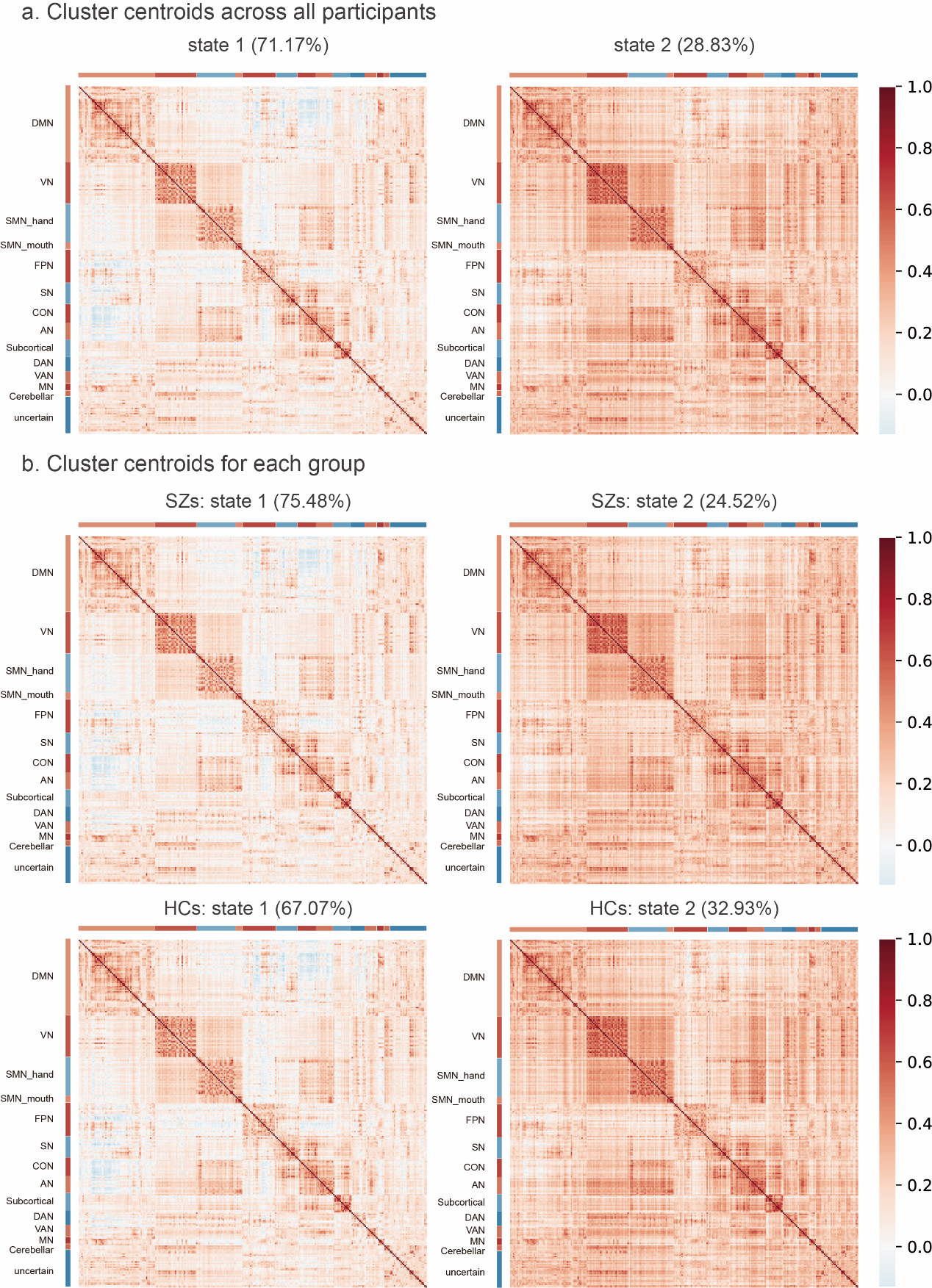
**Cluster centroids for each state across all participants (a).Cluster centroids for each state for each group (b). The color bar shows the strength of the functional connectivity between two brain nodes. For the abbreviations of brain networks, see Figure 1.


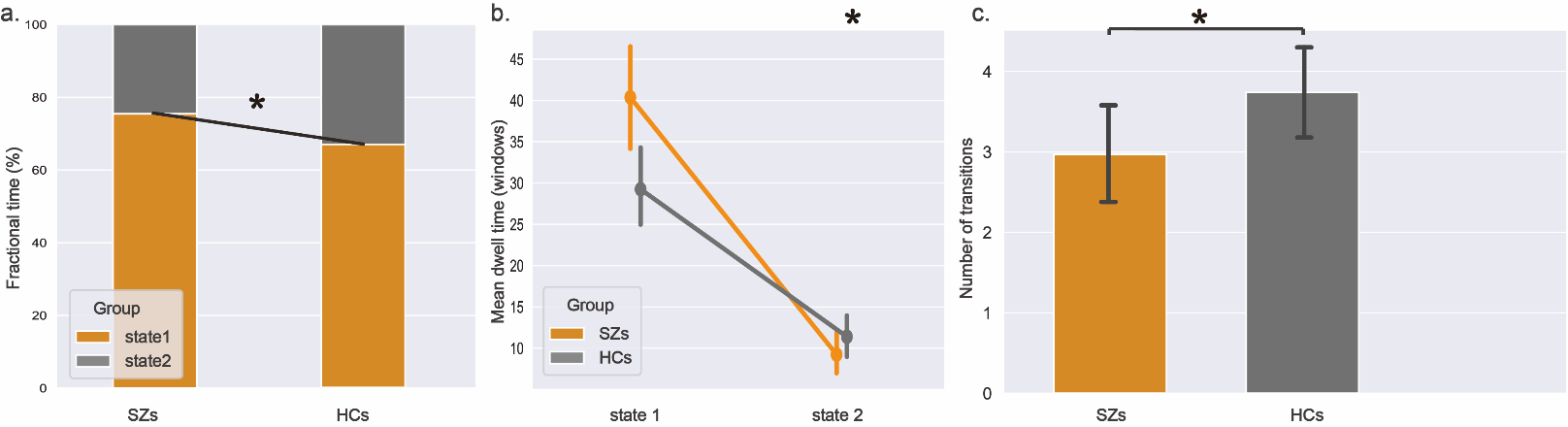
**Figure S3.** Comparison of state characteristics in patients and controls for validation. Patients with schizophrenia (SZs) showed altered fractional time in two states (a), decreased mean dwell time of state 2 (b), and decreased transitions between states (c) than healthy controls (HCs). * indicates false discovery rate corrected *P* value < 0.05.

**References**

1 Latora, V. & Marchiori, M. Efficient behavior of small-world networks. *Physical review letters* **87**, 198701, doi:10.1103/PhysRevLett.87.198701 (2001).

2 Rubinov, M. & Sporns, O. Complex network measures of brain connectivity: uses and interpretations. *Neuroimage* **52**, 1059-1069, doi:10.1016/j.neuroimage.2009.10.003 (2010).

3 Crucitti, P., Latora, V., Marchiori, M. & Rapisarda, A. Efficiency of scale-free networks: error and attack tolerance. *Physica A: Statistical Mechanics and its Applications* **320**, 622-642, doi:<https://doi.org/10.1016/S0378-4371(02)01545-5> (2003).

4 Lohmann, G. *et al.* Eigenvector centrality mapping for analyzing connectivity patterns in fMRI data of the human brain. *PloS one* **5**, e10232, doi:10.1371/journal.pone.0010232 (2010).
